# A survey of plant and algal genomes and transcriptomes reveals new insights into the evolution and function of the cellulose synthase superfamily

**DOI:** 10.1186/1471-2164-15-260

**Published:** 2014-04-04

**Authors:** Yanbin Yin, Mitrick A Johns, Huansheng Cao, Manju Rupani

**Affiliations:** 1 Department of Biological Sciences, Northern Illinois University, Montgomery Hall 325A, DeKalb, IL 60115-2857, USA

**Keywords:** Cell wall, CesA, CslH, CslD, Transcriptome, Ferns, Liverworts, CGA, Gymnosperms

## Abstract

**Background:**

Enzymes of the cellulose synthase (CesA) family and CesA-like (Csl) families are responsible for the synthesis of celluloses and hemicelluloses, and thus are of great interest to bioenergy research. We studied the occurrences and phylogenies of CesA/Csl families in diverse plants and algae by comprehensive data mining of 82 genomes and transcriptomes.

**Results:**

We found that 1) charophytic green algae (CGA) have orthologous genes in CesA, CslC and CslD families; 2) liverwort genes are found in the CesA, CslA, CslC and CslD families; 3) The fern *Pteridium aquilinum* not only has orthologs in these conserved families but also in the CslB, CslH and CslE families; 4) basal angiosperms, e.g. *Aristolochia fimbriata*, have orthologs in these families too; 5) gymnosperms have genes forming clusters ancestral to CslB/H and to CslE/J/G respectively; 6) CslG is found in switchgrass and basal angiosperms; 7) CslJ is widely present in dicots and monocots; 8) CesA subfamilies have already diversified in ferns.

**Conclusions:**

We speculate that: (i) ferns and horsetails might both have CslH enzymes, responsible for the synthesis of mixed-linkage glucans and (ii) CslD and similar genes might be responsible for the synthesis of mannans in CGA. Our findings led to a more detailed model of cell wall evolution and suggested that gene loss played an important role in the evolution of Csl families. We also demonstrated the usefulness of transcriptome data in the study of plant cell wall evolution and diversity.

## Background

Celluloses and hemicelluloses are the most abundant biopolymers in nature. In plants, they are the principal components of cell walls and the most promising renewable resources for producing biofuels [1,2]. The biosynthesis of celluloses and hemicelluloses is therefore one of the major research foci in plant biology. The past two decades have seen much progress in deciphering the molecular mechanisms of plant cell wall polysaccharide synthesis and regulation [3-8]. The identification of the cellulose synthase (CesA) gene family [9,10] and the CesA-like (Csl) gene families (collectively known as the CesA superfamily) [11] is one of the greatest achievements.

Early phylogenetic studies of CesA homologs in model plant organisms [11,12] established that there are eight Csl families: CslA, CslB, CslC, CslD, CslE, CslF, CslG and CslH, all belonging to the glycosyltransferase family 2 (GT2). Recent research in other flowering plants has added one more family (CslJ) [13]. It was proposed the Csl families might be involved in the synthesis of the backbones of hemicelluloses [11,14]. This “CSL hypothesis” has been strengthened by the functional characterization of CslA (mannan synthases) [15,16], CslC (xyloglucan synthases) [17], CslF (mixed-linkage glucan synthases) [18], and CslH (mixed-linkage glucan synthases) [19] genes. Although the functions of the other Csl families remain unknown, they are potentially involved in the synthesis of other cell wall polysaccharides or the same set of polysaccharides, e.g. through working together with other Csl or CesA genes [3].

The evolution of Csl families is also of interest to plant biologists studying the compositional diversity of cell walls [20-24]. Among the nine Csl families, CslA and CslC are distantly related to the other families; CslF and CslH are thought to be unique to monocots; CslB and CslG are confined to eudicots [25,26], and the rest of the families are found in both dicots and monocots [27]. Genomes of the lower land plants bryophyte moss *Physcomitrella patens* and lycophyte spike moss (*Selaginella moellendorffii*) only have representatives of the CesA, CslA, CslC and CslD families [27,28]. Six completed chlorophyte green algal genomes each have a single-copy CslA/C-like gene (herein named CslK), which represents the ancient CslA/C ortholog before a duplication happened in early land plants [27]. An evolutionary model was also proposed to explain the divergence order of Csl families, which has proved useful for our understanding of the cell wall diversity and evolution [22,24].

Over 40 plant genomes have been sequenced so far, including the first gymnosperm genome *Picea abies* (Norway spruce) [29]. However there is a lack of completed genomes for some key clades in the plant species tree: ferns, hornworts, liverworts, *Streptophyta* green algae (also known as advanced charophycean green algae, CGA), etc. Fortunately, the accumulation of transcriptome data in the GenBank database and the advent of the next generation sequencing have made a large amount of raw sequence data available for most of these key plants. For example, nine CGAs have significant amounts of transcriptome data recently available [30-32]. These data include ESTs (expressed sequence tags) sequenced by traditional Sanger technology, RNA-Seq data by the next generation 454 technology, as well as the pre-assembled UniGenes (mRNA contigs) in the Transcriptome Shotgun Assembly (TSA) sequence database; all of these data are available at the NCBI (National Center for Biotechnology Information) website.

Therefore, our goal in this study was to mine the transcriptomes and unfinished genomes of key plant species for Csl homologous genes in order to gain a better understanding of the evolution of the CesA/Csl superfamily. Specifically, we aimed to answer the question: when did each of the Csl families first appear in plants according to available sequence data? Answering this question will greatly improve our model of the evolution of Csl gene families and benefit the study of plant cell wall evolution and diversity.

## Results

### Csl genes in fully sequenced genomes: new findings

**Figure 1:**
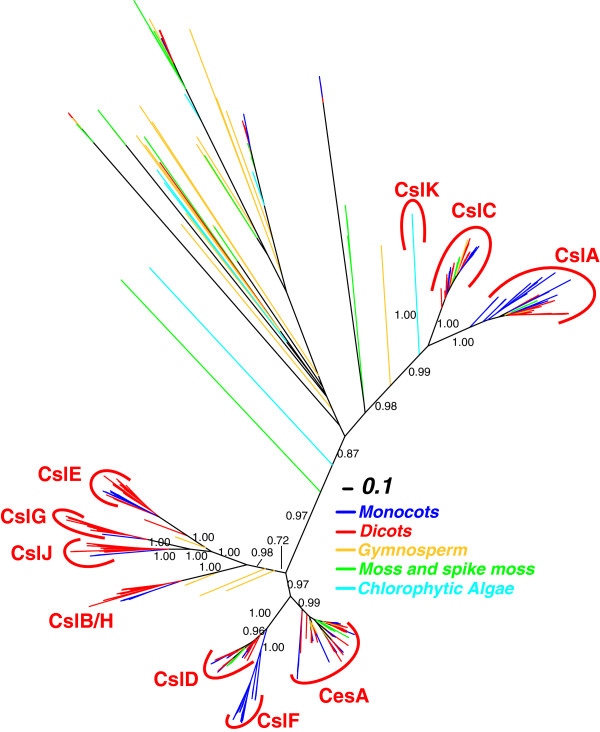
**Phylogeny of 893 GT2 proteins from 17 land plants and two green algae.** The full-length protein sequences were used to build the phylogeny. The FastTree bootstrap values (1.00 = 100%) larger than 0.70 are shown beside selected nodes forming the major Csl clusters. Csl clusters are labeled according to the presence of known Csl proteins in each cluster.

To retrieve Csl homologs, we scanned predicted protein sequences from the fully sequenced genomes of 32 land plants (23 dicots, six monocots, one gymnosperm, one moss and one spike moss), 10 CGA and two other algae (one *Glaucophyta* and one *Rhodophyta*), using two Pfam models (Cellulose_synt and Glycos_transf_2) as queries (see Additional file 1 for the list of species). The two Pfam models were used in our previous paper [27] and were able to retrieve all of the 39 documented *Arabidopsis* Csl genes. Figure 1 presents an unrooted phylogeny (protein IDs are provided in Additional file 2 and Additional file 3).

Compared with our previous work [27], one of the new findings is that CslG appears to have two member genes (Pavirv00027268m and Pavirv00027269m) from *P. virgatum* (see also Additional file 4), ~49% identical to AtCslG2 (AT4G24000.1). The grouping of these two switchgrass genes in CslG family is strongly supported (bootstrap value = 100%), suggesting that CslG can no longer be considered a dicot-specific family.

By including the newly sequenced *P. abies* genome in Figure 1 (orange color), we showed that gymnosperm proteins are found in CesA, CslA, CslC, CslD families. *P. abies* also has proteins clustered in the large CslB/H/E/J/G clade, but these proteins’ phylogenetic groupings are not well resolved. In later sections of this paper, more gymnosperm species with transcriptomes are included to better resolve the phylogenetic clustering of the gymnosperm Csl homologs.

The other finding concerns CslJ, which is close to CslG but is very well self-clustered (bootstrap value = 100%). CslJ was thought to be unique to cereals [25,26], but here it is shown to be widely present in four (sorghum, maize, foxtail millet and switchgrass) out of six fully sequenced monocot genomes and 16 out of 23 sequenced dicot genomes (Additional file 4).

The phylogeny of CslB and CslH shown in Figure 1 (also Additional file 4) suggests that these two families are so tightly clustered that they are hardly distinguishable. Therefore it might be more appropriate to consider them as a single family.

Figure 1 also includes other GT2 proteins. Between CslA/C/K and CesA/CslD/F/B/H/E/G/J, there are some loosely clustered groups that have very long branches. The long branches suggest that proteins in these clusters are quite different from each other; clusters with long branches are usually not very stable, a sign of small sample size or rapid sequence divergence.

To explore these clusters, we built a new phylogeny with proteins using fewer flowering plants and more algae (Additional file 5). Also included in the phylogeny are published CesA/Csl protein sequences from several non-plant species including brown algae, Oomycetes, fungi and bacteria [33].

In the new phylogeny (Additional file 5) most algal homologs form a large cluster (denoted as C) including two *Arabidopsis* (AT2G39630.1: dolichyl phosphase β-glucosyltransferase and AT1G20575.1: dolichol phosphate mannose synthase 1), two rice, and quite a few moss and spike moss GT2 proteins. A visual examination of the multiple sequence alignments in cluster C and those of the Csl families suggests that most of these non-Csl proteins do not have the characteristic ‘D,D,D,QXXRW’ motif typically found in Csl/CesA proteins. There are also many algal homologs clustered with the non-plant CesA/Csl proteins (aqua color), suggesting that they are likely to be CesA/Csl genes of distinct origin compared to the canonical land plant CesA/Csl genes. Further study including more non-plant Csl homologs will be needed in order to gain a better understanding of their origin and evolution.

### Mining for Csl homologs in short read transcriptomes/genomes

**Figure 2:**
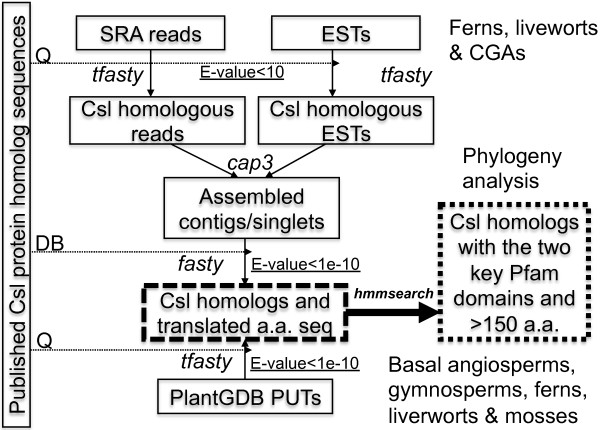
**Computational pipeline for data mining of short read transcriptome/genome data.** Details about the mined plants and algae are provided in Table 1 and 2. SRA: sequence read archive of the NCBI; ESTs: expressed sequence tags; *fasty* and *tfasty* are two homology search commands of the FASTA package [52] (see Methods); *hmmsearch* is a command of the HMMER3 package [47]. The two Pfam domains include Pfam models Cellulose_synt and Glycos_transf_2. PUTs are PlantGDB-assembled unique transcripts; Q means to use as the query set in the homology search; DB means to use as the database; published Csl protein homologs are from [27].

We developed a bioinformatics pipeline (Figure 2) that combined homology search and short read assembly to identify Csl homologs in: (i) transcriptome reads of nine CGAs and two ferns, (ii) genomic DNA reads of one liverwort, and (iii) pre-assembled uni-transcripts from GenBank ESTs, which consisted of PlantGDB-assembled unique transcripts (PUTs) from 26 plants, including six basal angiosperms, 16 gymnosperms (11 conifers, two cycads, one ginkgo, two gnetophyte), two ferns, one moss and one liverwort. Table 1 provides information about data for (i) and (ii) and Table 2 lists data for (iii).

**Table 1:** Short read sequence data sets of ferns, liverwort and CGAs

**Plant clades**	**Species**	**NCBI accessions**	**# of reads**	**References**
Fern	*Pteridium aquilinum*	SRX020701	730,579	[34]
Fern	*Ceratopteris richardii*	SRX154690	1,083,570	-
Liverwort	*Marchantia polymorpha*	SRX114614- SRX114615	300,372,599^a^	-
		SRX030759- SRX030787	22,854,396	-
*CGA: Charophyceae*	*Chara vulgaris*	SRX041525	740,355	[32]
	*Nitella hyalina*	SRX025843	949,065	[30,31]
*CGA: Coleochaetophyceae*	*Coleochaete orbicularis*	SRX017046	354,659	
	*Coleochaete_sp. CFD*	TSA contigs	18,386	
*CGA: Zygnemophyceae*	*Penium margaritaceum*	SRX025845	1,077,311	
	*Spirogyra pratensis*	SRX017045	614,139	
*CGA: Klebsormidiophyceae*	*Klebsormidium flaccidum*	SRX025847	994,649	
*CGA: Chlorokybophyceae*	*Chlorokybus atmophyticus*	SRX025846	444,743	
	*Chaetosphaeridium globosum*	SRX025844	884,238	

^a^Illumina reads, not used in this study.

**Table 2:** PlantGDB-assembled unique transcripts (PUTs)

**Plant clades**	**Species**	**# of GenBank ESTs**	**# of PUTs**
Gymnosperm	*Cryptomeria japonica*	57,720	24,299
Gymnosperm	*Picea abies*	14,619	8,715
Gymnosperm	*Picea engelmannii x Picea glauca*	28,190	13,880
Gymnosperm	*Picea glauca*	321,713	48,619
Gymnosperm	*Picea sitchensis*	206,402	31,215
Gymnosperm	*Pinus banksiana*	36,387	13,040
Gymnosperm	*Pinus contorta*	40,489	13,570
Gymnosperm	*Pinus pinaster*	35,139	15,648
Gymnosperm	*Pinus sylvestris*	76,256	73,609
Gymnosperm	*Pinus taeda*	329,066	72,829
Gymnosperm	*Pseudotsuga menziesii var. menziesii*	14,354	9,857
Gymnosperm	*Cycas rumphii*	22,000	10,901
Gymnosperm	*Zamia vazquezii*	11,495	7,657
Gymnosperm	*Ginkgo biloba*	21,709	10,210
Gymnosperm	*Gnetum gnemon*	10,756	6,193
Gymnosperm	*Welwitschia mirabilis*	10,137	6,606
Basal angiosperm	*Amborella trichopoda*	26,403	15,772
Basal angiosperm	*Aristolochia fimbriata*	16,454	7,967
Basal angiosperm	*Liriodendron tulipifera*	24,146	14,232
Basal angiosperm	*Nuphar advena*	20,601	13,789
Basal angiosperm	*Persea americana*	16,620	10,928
Basal angiosperm	*Saruma henryi*	10,281	6,754
Liverwort	*Marchantia polymorpha*	33,764	10,959
Moss	*Syntrichia ruralis*	10,010	7,087
Fern	*Adiantum capillus-veneris*	30,561	16,944
Fern	*Ceratopteris richardii*	5,186	4,234

In Figure 2, the dashed rectangle contains all the Csl-homologous peptides in the surveyed transcriptome/genome. The peptide sequences were translated from assembled nucleotide contigs and singletons according to the *fasty* alignment with their best Csl hits, which are published Csl proteins previously classified into the 10 existing Csl families [27]. Note that for PlantGDB’s PUTs, the assembly step was not needed and *tfasty* was used to derive the translated peptide sequences.

Given that transcriptome sequencing and subsequent assembly are unlikely to recover the full-length transcripts, it was not surprising that many Csl homologs in the dashed rectangle of Figure 2 were short fragments. To clean the data, we applied the following filters to keep significant and long Csl homologs (Figure 2, dotted rectangle): (i) they had to be highly similar to known Csl proteins (E-value < 1e-10); (ii) they had to match the two characteristic Pfam domains (Cellulose_synt and Glycos_transf_2, E-value < 1e-2); and (iii) they had to be longer than 150 amino acids. These filters tend to be very stringent, so that a Csl homolog that passed all of the three filters would very likely be a true Csl gene. The filters were also helpful in reducing the impact of contamination or low quality reads on our downstream phylogenetic analyses. In particular, we found that the second filter was very critical for removing false positives. However, we were very flexible about the length filter because we did not want to miss real orthologs. When necessary, we manually inspected peptides shorter than 150 a.a. to select and include appropriate ones in the phylogenetic analyses.

For phylogenetic analysis, we combined Csl-homologous peptides of a specific plant clade (e.g. CGAs) with the known Csl proteins and then generated new phylogenies. The new phylogenies were then examined to determine if the new homologs clustered with the existing Csl families or if they formed distinct new clusters.

### CGAs have representative genes from CesA, CslC and CslD families

**Figure 3:**
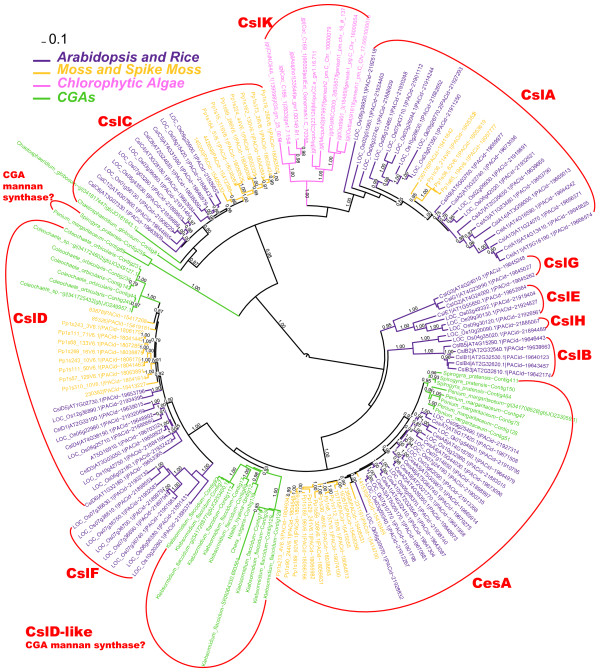
**Phylogeny with CGA homologs.** Sequences of this phylogeny include 52 CGA peptides longer than 200 a.a. Other proteins are 131 pre-classified Csl proteins selected from Additional file 5.

Figure 3 presents a phylogeny with CGA homologs longer than 200 a.a. and Additional file 6 provides the sequences. It is clear that CGA homologs are found in the CslC, CslD and CesA clusters. It is surprising however that none are found in the CslA cluster, as CslA enzymes are responsible for the synthesis of mannans, which have been found in the cell walls of CGAs [35]. We investigated whether any true CslAs were removed in the stringent filtering steps by manually inspecting the *fasty* search results, and found no false negatives.

Penium_margaritaceum-Contig85 is the only CGA peptide that has a known CslA protein (Os02g09930.1) as the best hit (identity = 24%). However, it is not clustered within the CslA clade but with another CGA peptide (Spirogyra_pratensis-Contig255) with identity = 63%. This Spirogyra_pratensis peptide has Os03g56060.1 of CslC as the best hit (identity = 27%). These two CGA peptides are further placed basal to CslA, CslC and CslK clusters (Figure 3).

Lowering the length filter to 100 a.a. did not find any shorter peptides that clustered within the CslA clade (Additional file 7). However, three more peptides (Chara_vulgaris-Contig143, Nitella_hyalina-SRR064326.525840 and Nitella_hyalina-SRR064326.70219) clustered with the CslC clade, indicating that four out of the nine surveyed CGA species have CslC proteins.

CslD and CesA families both have CGA homologs. CslD homologs were found in *Coleochaete* species, while CesA homologs were found in *Spirogyra pratensis* and *Penium margaritaceum* (Figure 3). We also tried to include peptides shorter than 200 a.a. and found CesA homologs in *Klebsormidium flaccidum* and CslD homologs in *Chaetosphaeridium globosum* (Additional file 7). Deeper RNA sequencing will be needed to resolve the question of whether CslD and CesA genes are present in the other CGA species.

The presence of a major CGA-specific cluster close to the CslD clade, containing peptides from *Klebsormidium flaccidum*, *Nitella hyaline* and *Chara vulgaris* (Figure 3), is highly interesting. If peptides shorter than 200 a.a. were included, more CGA sequences would be clustered within this CslD-like clade (Additional file 7). It is possible, but highly speculative, that this CGA-specific clade encodes the missing CGA mannan synthases. More data are needed to determine if this clade is actually part of the CslD family or represent a new Csl family.

### Liverworts have representative genes from CesA, CslA, CslC and CslD families

The model liverwort species *Marchantia polymorpha* has 33,692 ESTs and 31 genomic DNA datasets in the SRA database of NCBI. The EST data has been assembled into PUTs in the PlantGDB. Among the 31 SRA datasets, 29 are from 454 sequencing, which yields longer reads than Illumina, so we used these 29 datasets, a total of 13GB. We identified liverwort Csl homologs by combining all sequences together and using the protocol shown in Figure 2.

**Figure 4:**
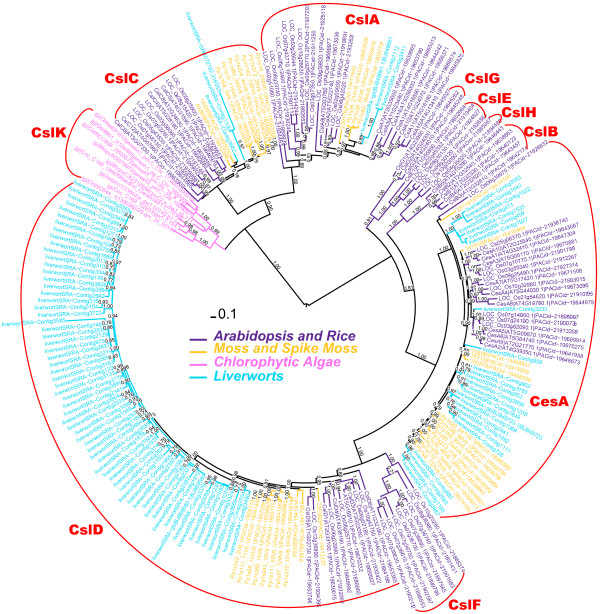
**Phylogeny with liverwort homologs.** Sequences of this phylogeny include 96 liverwort peptides, most of which longer than 200 a.a. Three shorter proteins were added and clustered within the CslA cluster.

Figure 4 shows a phylogeny with liverwort homologs longer than 200 a.a. and a small number of selected homologs between 100 and 200 a.a. Additional file 8 provides the sequences. Similar to the fully sequenced moss and spike moss genomes, liverwort appears to have genes in CslA, CslC, CslD and CesA clusters but not in the CslB/H/E/G clusters. It is also clear that liverwort Csl homologs are often clustered with moss and spike moss sequences and ancestral to their corresponding orthologs in seed plants, suggesting that the sequence diversification of these genes happened after the split of liverworts and seed plants. Compared to other Csl families, CslD seems to have many more liverwort homologs. However, many of these homologs are very similar to each other (identity > 95% at the nucleotide level and also demonstrated by the very short branch lengths), which might be due to under-assembly. Hence the actual number of CslD homologs in liverwort is probably much lower. Since our goal is not to quantitatively but qualitatively assess the occurrence of Csl families in different plants, such under-assembly does not affect any of our conclusions. There are three expressed liverwort homologs found in the PlantGDB’s PUTs (see below), each in the CslC, CslD and CesA clusters respectively.

### Ferns have representative genes from CesA, CslA, CslC, CslD, CslE, CslB and CslH families

Two fern species, *Ceratopteris richardii* and *Pteridium aquilinum*, have transcriptome data sequenced by 454 in the NCBI SRA database (Table 1). *C. richardii* and *Adiantum capillus-veneris* also have ESTs, which are assembled into PUTs in PlantGDB (Table 2). Following the procedure shown in Figure 2, we identified Csl homologs in the three fern species.

**Figure 5:**
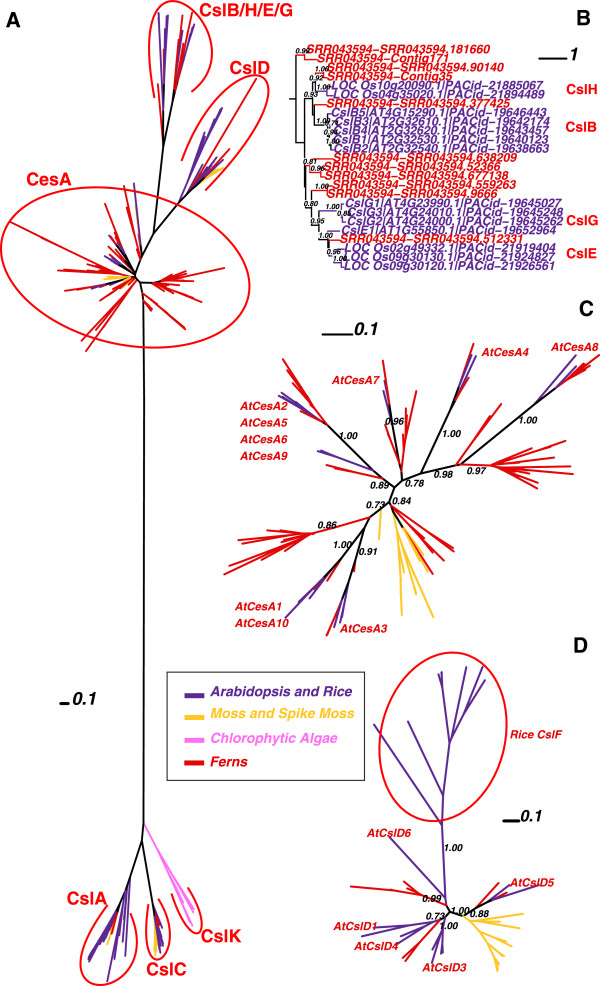
**Phylogenies with fern homologs.** Sequences of these phylogenies include 182 fern peptides longer than 150 a.a. **(A)** The phylogeny contains all sequences; **(B)** The phylogeny shows only the CslB/H/E/G cluster; **(C)** The phylogeny shows only the CesA cluster; **(D)** The phylogeny shows only the CslD cluster. Sequences in **(B)**, **(C)** and **(D)** were extracted from **(A)** and then re-aligned; the phylogeny was then recomputed based on the new alignments.

The phylogeny shown in Figure 5A includes fern peptides longer than 150 a.a. Additional file 9 provides the sequences. Ferns have representative genes in CslA, CslC, CslD, CesA, and even in the CslB/H/E/G clusters. Of the three fern species, *C. richardii* and *A. capillus-veneris* have homologs only in the CesA and CslD clusters while *P. aquilinum* has genes in all the other Csl clusters. In fact, *C. richardii* has many fewer Csl homologs than *P. aquilinum* (15 vs. 281; length > 100 a.a.), although the former has many more reads in the surveyed datasets (Table 1). This suggests that the transcriptome data of *C. richardii* might be very biased and does not capture the transcripts of many Csl genes, as it is unlikely that its genome does not encode CslA and CslC genes.

Most interestingly, *P. aquilinum* homologs are evidently found in CslE, CslB and CslH clades (Figure 5B). The fern CslB ortholog SRR043594-SRR043594.377425 has AtCslB2 (AT2G32540.1) as its best hit (sequence identity = 48% at the a.a. level) among all known Csl proteins; the CslH ortholog SRR043594-Contig35 matches OsCslH1 (Os10g20090.1) as the best hit (sequence identity = 36%); and the CslE ortholog SRR043594-SRR043594.512331 has Os09g30130.1 of the CslE family as the best hit (sequence identity = 60%). There are also fern homologs phylogenetically basal to both CslB and CslH. This suggests that the CslB/H/E clades had already diverged before ferns appeared, possibly through ancient duplications from older Csl families (i.e. CesA or CslD). Given that the completed spike moss genome does not have CslB/H/E genes, the emergence of these Csl families must have happened after spike moss split from more advanced vascular land plants but certainly before ferns.

The CslD family seems to have already diversified before ferns split from seed plants, as AtCslD1/4 and AtCslD5 have clear orthologs in ferns (Figure 5D). Similarly, different CesA sub-clusters containing the *Arabidopsis* genes CesA3, CesA4, CesA7, CesA8, CesA1/10 and CesA2/5/6/9, respectively, all have orthologs in ferns (Figure 5C), suggesting that their divergence occurred as early as in the last common ancestor of ferns and later evolved land plants. It is most interesting to observe that, for the three major components of the cellulose synthase complex of secondary cell walls in *Arabidopsis*: CesA4, CesA7 and CesA8, their common ancestral genes had already diversified in ferns, in contrast to the earliest vascular plant spike moss, whose CesAs are all clustered into one monophyletic group (yellow color).

There are also additional fern-specific CesA clusters, e.g. the large red clusters in the CesA circle of Figure 5A and also the red sub-clusters beside AtCesA8 and beside AtCesA1/10 of Figure 5C. All the members of these clusters have known CesA proteins as the best hit, but future experimental studies are needed to verify whether they truly have cellulose synthase activity or not.

### Gymnosperms have Csl genes basal to CslB/H and to CslE/G respectively

Although there is one gymnosperm genome *P. abies* available, Figure 1 suggests that more gymnosperm sequences are needed to resolve the uncertain clustering of *P. abies* homologs in CslB/H/E/J/G clusters. Therefore, we selected from PlantGDB six basal angiosperms, 16 gymnosperms, two ferns, one moss and one liverwort (Table 2). The basal angiosperms include plant species that are neither eudicotyledons nor monocotyledons, such as magnoliids, which are ancestral to both dicot and monocot plants. Following the procedure in the bottom part of Figure 2, we identified Csl homologs in these plants.

**Figure 6:**
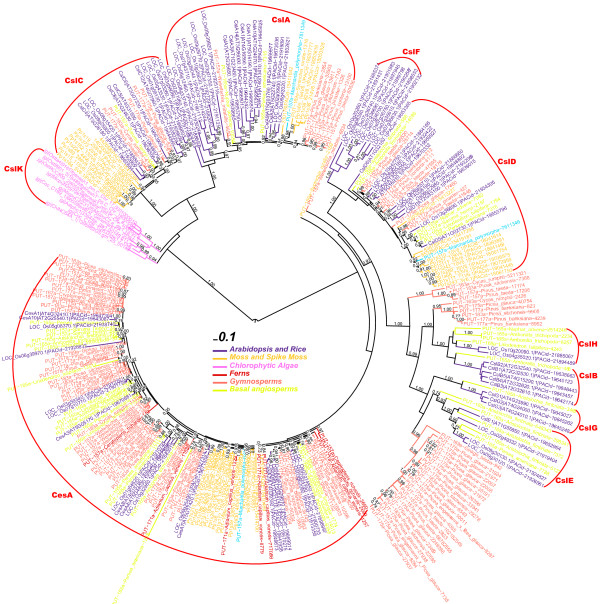
**Phylogeny with PUT homologs.** Sequences of this phylogeny include 167 PUT peptides longer than 200 a.a.

Figure 6 shows a phylogeny with all of the Csl homologs longer than 200 a.a. from the 26 surveyed plant transcriptomes and Additional file 10 provides the sequences. We mainly looked at gymnosperms and basal angiosperms. In agreement with what we found in Figure 1, no gymnosperm homolog (lighter red) is found inside the individual cluster of CslB/H/E/G. More precisely, the CslE family clustered with a large gymnosperm cluster with a bootstrap value lower than 70%. So it appears that each individual family does not have clear orthologs in gymnosperms. Instead, there are gymnosperm-specific clades basal to CslE/G families and CslB/H families, respectively. Basal angiosperm homologs however were found in all of the individual families including CslG. Interestingly, all of the six basal angiosperms have genes in the CslB/H/E/G clusters. Particularly, *Aristolochia fimbriata* has six genes found in all of the four Csl families.

## Discussion

To our best knowledge, this is the first comprehensive and large-scale data mining of Csl homologs in the transcriptomes of various plants and algae. Prior to our study, Sorensen et al. searched the EST data of *C. nitellarum* (CGA species) and found CslD orthologs [35]. Richmond built a web resource (http://cellwall.stanford.edu) in 2000 to collect Csl genes in plant genomes and ESTs [11], but that web resource is no longer available. Publications of transcriptome/genome data of diverse plants and algae (Tables 1 and 2) in the past few years have made our comprehensive search possible.

Here we categorized CesA/Csl genes residing in 44 fully sequenced plant and algal genomes (Additional file 11), as well as 38 transcriptomes of CGAs, ferns, gymnosperms, and basal gymnosperms, and raw genomic DNA reads from liverworts (Additional file 6, Additional file 8, Additional file 9, Additional file 10). We studied their distribution in 10 different Csl protein families using phylogenetic analyses, which not only offer cell wall polysaccharide and bioenergy researchers with a list of Csl genes in bioenergy-related crops, but also provide new insights into the evolution and function of the CesA/Csl families in different plants.

### About mannans in CGAs

**Figure 7:**
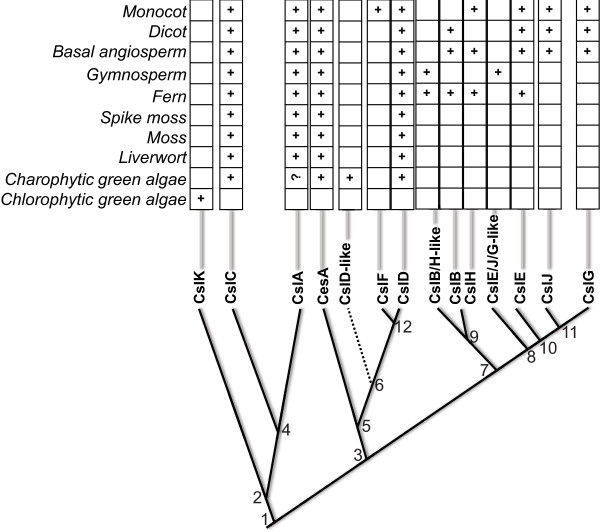
**An evolutionary model of Csl gene families.** The top panel shows the occurrence of Csl families. “+” means the family is found; “?” means it is uncertain. In the bottom panel, the numbers 1 to 12 are used to label each divergence node, which are detailed in the main text. The dotted line means it is uncertain if the CslD-like family truly exists.

As shown in Figures 3 and 7, CslA appears to be absent in CGAs. There are many possible reasons for why these genes are missing in CGAs, but we believe the following are the most likely: (i) CGA mannan synthase genes were not captured by the transcriptome data that we mined due to low expression, or (ii) CGA mannan synthases are not encoded by the canonical CslA gene family. In other words, convergent evolution may have given gene families other than CslA the ability to synthesize mannans in CGAs, e.g. the CGA-specific clusters found in Figure 3. Notably, these clusters are close to the CslD family in the phylogeny. The literature contains discussions of the possibility of CslD proteins are glucomannan synthases [3,36,37]. It is therefore tempting to speculate that the CslD and/or CslD-like genes (Figure 3 and Additional file 7) are responsible for the synthesis of CGA mannans, given that (i) CGAs have genes only in the CesA, CslA, CslD and CslD-like clusters, (ii) the cell walls of CGAs contain both xyloglucans and mannans, (iii) the CslA family encodes xyloglucan synthases and (iv) the function of the CslD family is still unknown.

The first possibility could be validated by deeper RNA sequencing of CGAs that have experimental evidence of mannans, e.g. *Coleochaete nitellarum* and *Spirogyra spI*[35]*.* However, we noticed that two closely related species, *Coleochaete orbicularis* and *Spirogyra pratensis*, were included in our data mining and only one relevant sequence (Spirogyra_pratensis-Contig255) was found in either species; this sequence is most similar to a known CslC protein. For this reason, the possibility that mannan synthesis genes are missing from the data for artifactual reasons seems unlikely.

### About MLG in horsetail (*Equisetum arvense*)

In NCBI’s taxonomy database, the two fern species, *Ceratopteris richardii* and *Pteridium aquilinum*, both belong to *Polypodiopsida* under *Moniliformopses* (ferns). Interestingly, horsetails (*Equisetopsida*) that are also of *Moniliformopses* have been shown to have mixed-linkage glucans (MLGs) in their cell walls [38-41]. Since MLGs are only narrowly found in the plant kingdom, in *Poaceaes*, horsetails and some algae, it has been proposed that horsetails and algae might have independently acquired their abilities to synthesize MLGs by using enzymes of the Csl families. Here we found that CslH has orthologs in the fern species *Pteridium aquilinum*. It is likely that these CslH genes also encode MLGs in *Pteridium aquilinum*, although experimental evidence is needed to prove this. On the other hand, this suggests that horsetails probably also have CslH orthologs that are responsible for the synthesis of MLGs in their cell walls. Therefore, our finding supports the hypothesis that CslH genes were in the common ancestor of ferns and seed plants but later lost in gymnosperms. Such gene loss event might be fairly prevalent, as *Ceratopteris richardii*, which has more reads than *Pteridium aquilinum* (Table 1), appears to have no CslH genes.

Given that both CslF and CslH encode MLG synthases and CslF is strictly confined to monocots, we conclude that CslH is the more ancient MLG synthase family [41].

It was suggested that leafy liverwort *Lophocolea bidentata* might have MLG-like polysaccharides [42], but our search in the liverwort *Marchantia polymorpha* genome did not find any CslH orthologs. Spike moss and CGAs were also suggested to have MLG [35], but the MLG is unlikely to be synthesized by CslH as no CslH orthologs were found in the completed spike moss genome or the surveyed CGA transcriptomes. In this case, the convergent evolution hypothesis is still a plausible explanation for the synthesis of MLGs in these organisms that do not have CslH and CslF families.

### Evolution of Csl families

Many of our previous views about the Csl families were changed in light of our new findings, which led to a revised evolutionary model with more details (Figure 7). Twelve nodes were labeled to represent the speculated evolutionary events that might have led to the divergence of CesA/Csl gene families.

#### About CslA/C/K

Node 1 represents the endosymbiosis event(s) that gave rise to the earliest plant cell. Two distinct ancestral genes were passed to the earliest plant cell, which shared an even earlier GT2 ancestor in ancient prokaryotes. One gene was the ancestor of extant CslA/C/K families and the other was the ancestor of the rest of the Csl families. A larger scale analysis including non-plant GT2 proteins will be useful to disentangle the different origins of the two groups of families.

After node 2, the ancestral gene became the current CslK family in chlorophytes, while in CGA, it evolved into the CslA and CslC families through duplication (node 4). This duplication event should have occurred after the split of CGAs and chlorophytes. We did not find CslA genes in CGAs, suggesting that CslA might have been lost in evolution.

#### About CesA/CslD/F

The other ancestral gene that the earliest plants inherited was very likely to be a CesA gene, which might be from some ancestral cyanobacteria [43,44]. Node 3 represents an early gene duplication that occurred, probably in ancient algal species, where one gene later evolved to be the latest common ancestor of CesA/CslD/F families, while the other evolved to be the latest common ancestor of CslB/H/E/J/G.

Node 5 implies that the ancestor of CesA/CslD duplicated and diverged into the CesA and CslD clades. As both families are present in CGAs, their divergence must have happened before CGAs appeared. Afterwards, the evolution of the CslD genes seem to be enigmatic in that some CGAs (*Coleochaete orbicularis* and *Chaetosphaeridium globosum*) have CslD homologs while others have CslD-like homologs (Figure 3 and Additional file 7). It is therefore uncertain if there was an additional divergence (node 6) that gave rise to the CslD-like homologs in CGAs. Completed CGA genomes will be needed to reach a conclusive answer.

It is worthy of mentioning that different CesA subfamilies, including the AtCesA4, AtCesA7 and AtCesA8 subfamilies, diversified after spike moss but before ferns appeared (Figure 5C). This suggests that the secondary cell wall cellulose synthase protein complex has been in existence since ferns.

CslF genes are only present in monocots, and they have long branches in the phylogenies (Figure 1), suggesting a rapid divergence after splitting from CslD family through duplication (node 12). Therefore, among all Csl families, CslF was the last one to arise.

#### About CslB/H/E/J/G

The divergence of CslB/H and CslE/J/G (node 7) should be much later than node 5, because CslB/H/E appeared since ferns. The common ancestor of CslB/H/E/J/G might be very ancient, but the diversification of this ancestral gene into each of the individual families seems to have occurred much later.

**Figure 8:**
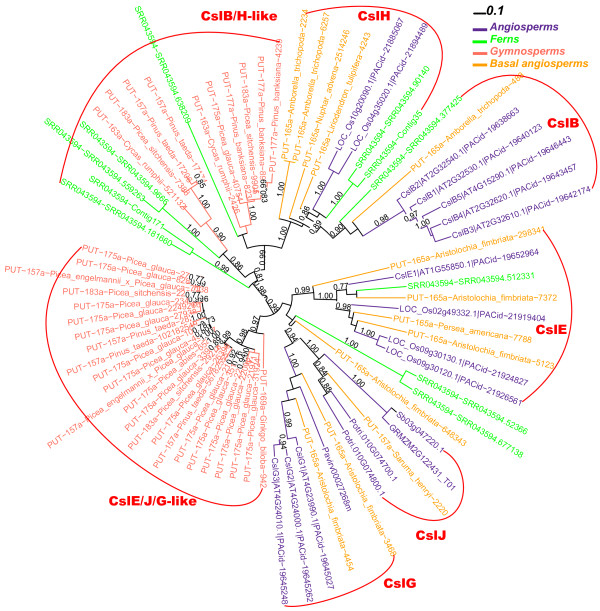
**Phylogeny with CslB/H/E/J/G homologs from selected organisms.** Sb03g047220.1, GRMZM2G122431_T01, Potri.010G074700.1, Potri.010G074800.1 of CslJ family and Pavirv00027268m of CslG family are selected from Figure 1. The rest proteins are selected from Figures 3 to 6.

The most exciting findings of this paper are about these families. Prior to our study, it was believed that: i) CslB and CslG are dicot-specific; ii) CslH and CslJ are found only in cereals; iii) CslE is only found in angiosperms. As shown in Figure 8, these views are subject to modifications: 1) CslB, CslH and CslE have orthologs in ferns and basal angiosperms; 2) CslG is found in switchgrass and basal angiosperms (also see Figure 1 and Additional file 4); Additional file 3) CslJ is found in most dicots (Figure 1 and Additional file 4) and in basal angiosperms too.

No gymnosperm genes are found in any of the individual families of CslB/H/E/J/G. However the presence of CslB/H-like cluster (Figure 8) suggests that CslB and CslH might have evolved in ferns and then lost in gymnosperms. Similarly the gymnosperm-specific CslE/J/G-like gene cluster contains expressed genes from almost all surveyed gymnosperms including the ginkgo species, suggesting that it is functionally very important and conserved (short branches in Figure 8).

It remains a mystery why and how gymnosperms lost CslB, CslH and CslE genes but retained the apparently more ancestral CslB/H-like and CslE/J/G-like families. However, all of the CslB/H/E/J/G families are no longer narrowly distributed and they appear to be much older than previously thought. It has been suggested that gymnosperms have lower substitution rate in their genomes [45], which should be considered for the future study of the evolution of CslB/H/E/J/G families.

About the divergence order of these families, node 8 and 9 must precede the occurrence of ferns. CslE then diverged from CslJ/G (node 10), probably also before ferns. CslG might have evolved in early angiosperms through gene duplication from the CslJ family (node 11).

Our study suggests that gene duplication and gene loss (e.g. loss of CslB/H/E in gymnosperms) occurred very often throughout plant genome evolution, and together they have played a significant role in shaping the expansion and diversification of the Csl families.

## Conclusions

In summary, the following major contributions were made in this paper: 1) we demonstrated that the toolkits for the study of the plant cell wall evolution and diversity could be complemented by bioinformatics data mining of the transcriptomes of plant clades that do not have completed genomes; 2) we found that fern transcriptomes have expressed genes of the CslB/H/E families so these families are much older than we thought; 3) we predicted that CslH genes might also exist and encode MLG synthases in horsetails; 4) we speculated that the mannan synthases in CGAs might be encoded by Csl families other than CslA as it is missing in all surveyed CGA transcriptomes; and 5) we proposed a more complete model for the evolution of Csl families and suggested that gene loss following duplication played a significant role in the evolution of Csl gene families.

## Methods

### Sequence data

Previously categorized Csl protein sequences were downloaded from the supplemental data of [27].

The fully sequenced plant and algal genomes were downloaded from Phytozome and JGI [46], except for *Picea abies*, downloaded from http://congenie.org. The HMMER3 package [47] was used to search the two Pfam domains (Cellulose_synt and Glycos_transf_2) against the above genomes, following our previous papers [27,48,49]. The fern, liverwort and CGA short read data sets were downloaded from the NCBI SRA database. The pre-assembled PUT data sets were downloaded from PlantGDB [50].

The owners of the unpublished fern (*Ceratopteris richardii*) transcriptome data and the unpublished liverwort (*Marchantia polymorpha*) genome data agreed with the use of these data in this study and were acknowledged in the Acknowledgement. The liverwort genome sequence data were produced by the US Department of Energy Joint Genome Institute (http://www.jgi.doe.gov/) in collaboration with the user community. All other SRA sequence data that have been published were properly cited in Table 1.

### Data mining pipeline

The pipeline was depicted in Figure 2. For the assembly of 454 transcriptomes/genomes of ferns, liverwort and algae, we used *cap3*[51] with overlap length > 60 bp and overlap percent identity > 97% (-o 60 and -p 97). Because short read assembly is well known to be computationally intense when the data size is large, we did a pre-screening homology search prior to the assembly and only assembled the reads of a same species that are homologous to known Csl proteins.

The FASTA package [52] was used for all homology searches. Specifically, *fasty* and *tfasty* commands were used, which have the advantage that they can tolerate sequence errors and tend to yield longer alignments by including stop codons and frame shifts, as compared to the common BLAST searches.

After *fasty/tfasty* search, the peptide sequences were translated from assembled nucleotide contig/singleton sequences according to the alignment with their best Csl hits. Symbols of frame shifts (“/” and “\”) and stop codons (“*”) in the *fasty/tfasty* alignments were removed before multiple sequence alignment (MSA).

### Phylogenetic analysis

MSAs were generated using MAFFT v6.935b with the L-INS-i method [53], which is among the most accurate sequence alignment algorithms. Phylogenies were made using the FastTree program version 2.1.3 [54]. FastTree implements an ultrafast and fairly accurate approximate maximum likelihood method. The accuracy of FastTree is considered to be slightly better than PhyML version 3.0, with minimum-evolution nearest neighbor interchanges moves, and is 100 to 1,000 times faster and requires much less computer memory.

FastTree analyses were conducted with default parameters; specifically, the amino acid substitution matrix was JTT, the number of rate categories of sites (CAT model) was 20, and the local support values of each node were computed by resampling the site likelihoods 1000 times and performing the Shimodaira Hasegawa test. Based on our previous work [27,48,49], FastTree performs sufficiently well for protein family evolution studies.

We also tried the much slower but more accurate PhyML program to build all of the phylogenies and the tree topology does not differ much and does not change any of our findings. The iTOL server was used to generate the phylograms [55].

### Availability of supporting data

The data sets supporting the results of this article are included within the article and its additional files.

## Competing interests

The authors declare that they have no competing interests.

## Authors’ contributions

YY conceived this study, carried out all the analysis and wrote the paper. MAJ, HC, and MR helped in the data analysis and the paper writing. All authors read and approved the final manuscript.

## Supplementary Material

Additional file 1 44 fully sequenced plants and algae. Click here for file

Additional file 2 **Circular view of Figure **1**.** Click here for file

Additional file 3 **Sequences included in Figure **1**.** Click here for file

Additional file 4 **Zoomed-in view of CslB/H/E/J/G clusters in Figure **1**.** Click here for file

Additional file 5 Phylogeny with plant and algal GT2 homologs forming three large clusters (the inset shows the radial view). Click here for file

Additional file 6 Csl homologs found in CGAs and sequences. Click here for file

Additional file 7 Phylogeny with CGA homologs longer than 100 a.a. Click here for file

Additional file 8 **Csl homologs found in *Marchantia polymorpha *** **and sequences.** Click here for file

Additional file 9 Csl homologs found in ferns and sequences. Click here for file

Additional file 10 Csl homologs found in PUTs of surveyed plants and sequences. Click here for file

Additional file 11 Csl homologs of 44 plant and algal genomes. Click here for file
