# Supplementary figures and images for: A survey of plant and algal genomes and transcriptomes reveals new insights into the evolution and function of the cellulose synthase superfamily
Source: BMC Genomics. 2014 Apr 4;15:260. doi: 10.1186/1471-2164-15-260 (PMC4023592; doi:10.1186/1471-2164-15-260)

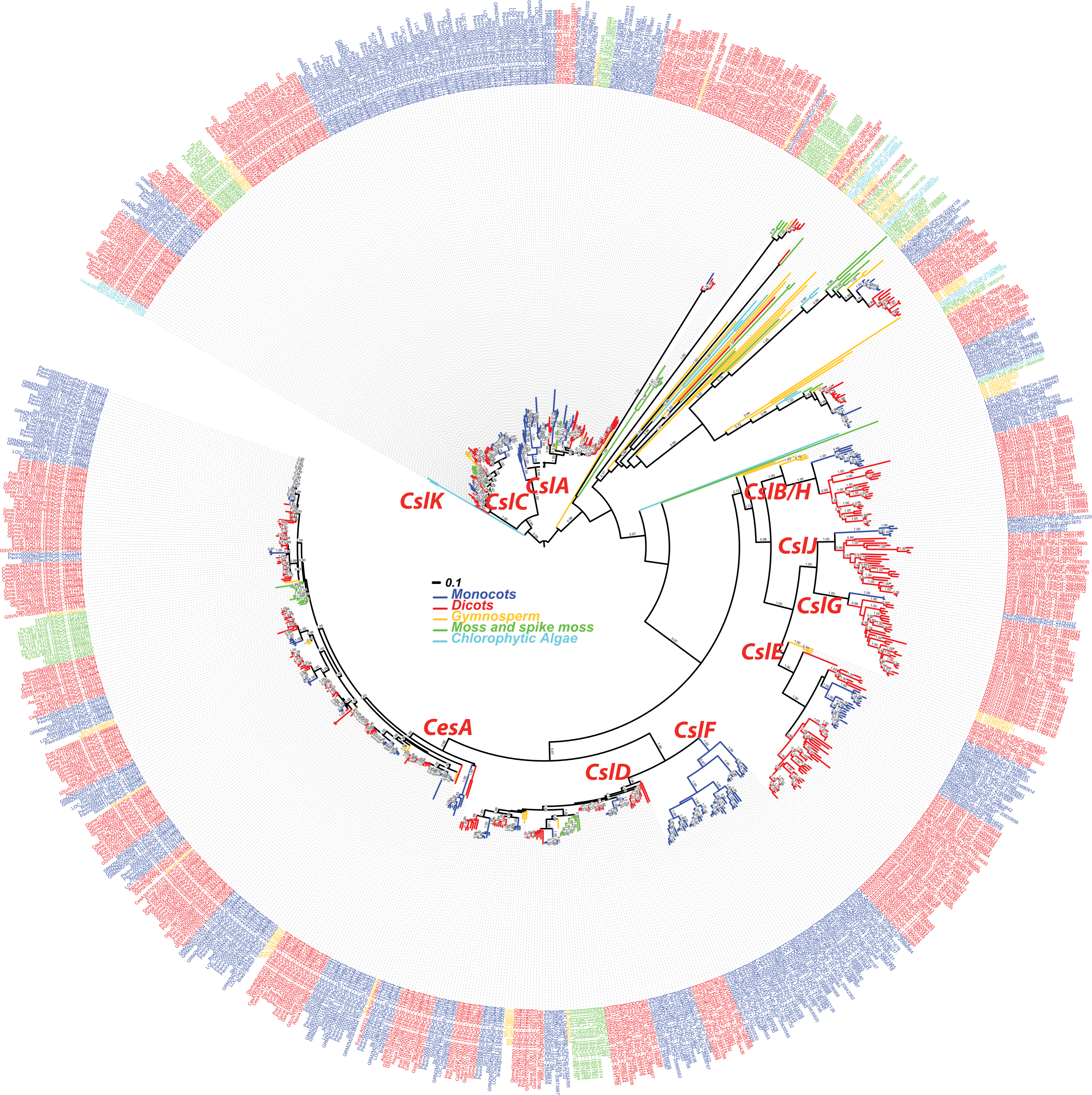

Supplement: Additional file 2 — Circular view of Figure 1. [file 1471-2164-15-260-S2.pdf]

# CsIB/H

**CslJ**

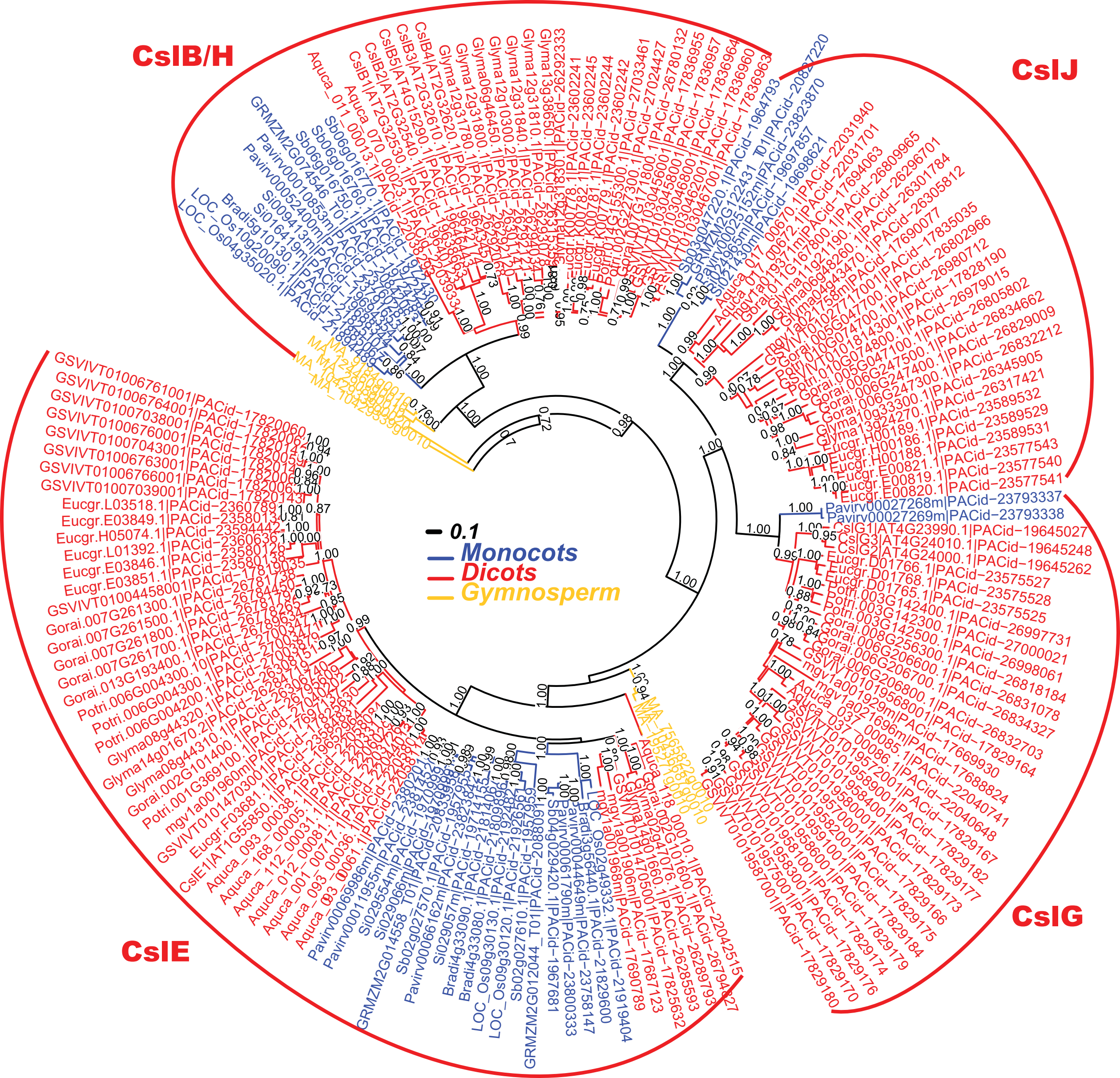

**CsIE**

**CslG**

Supplement: Additional file 4 — Zoomed-in view of CslB/H/E/J/G clusters in Figure 1. [file 1471-2164-15-260-S4.pdf]

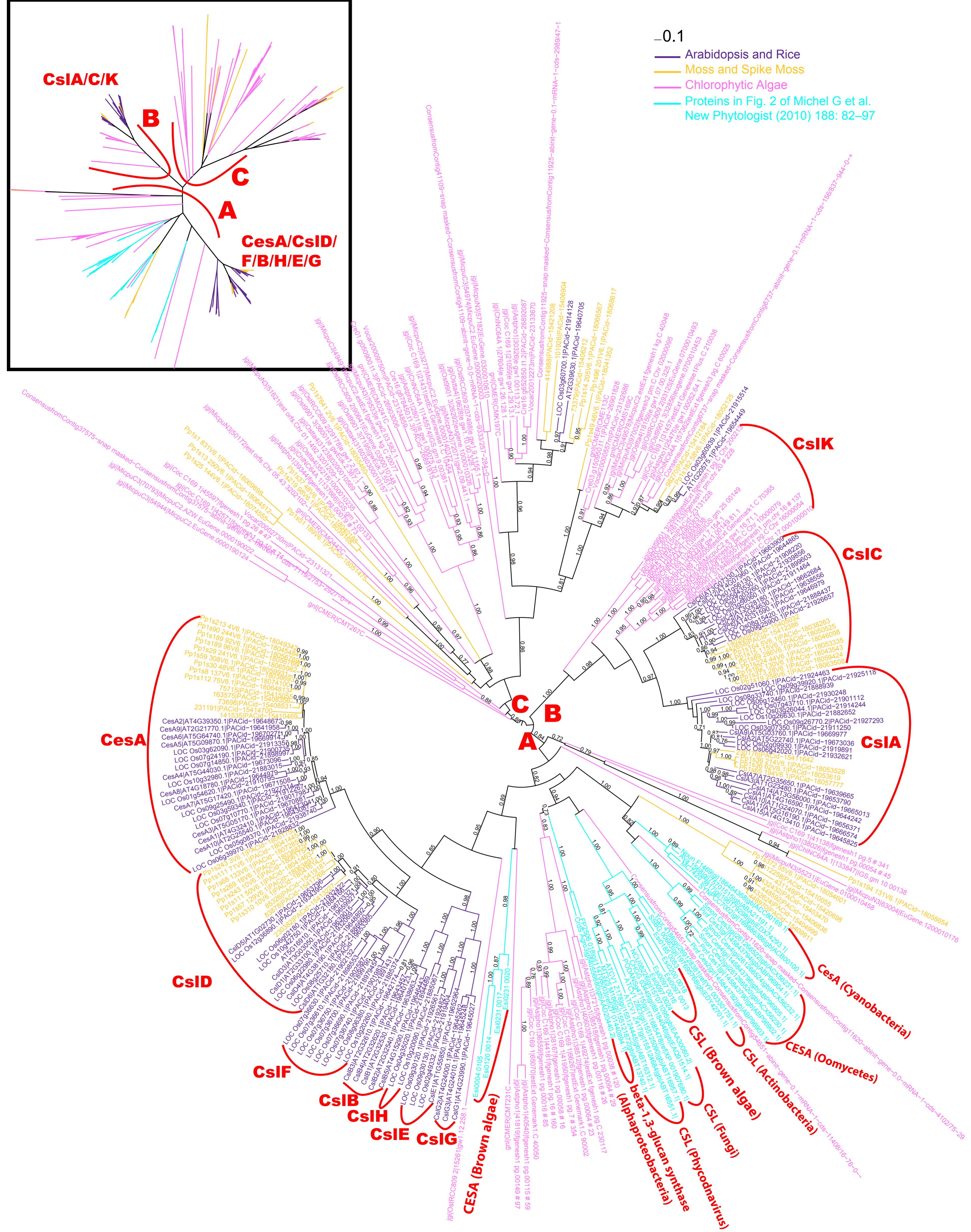

Supplement: Additional file 5 — Phylogeny with plant and algal GT2 homologs forming three large clusters (the inset shows the radial view). [file 1471-2164-15-260-S5.pdf]

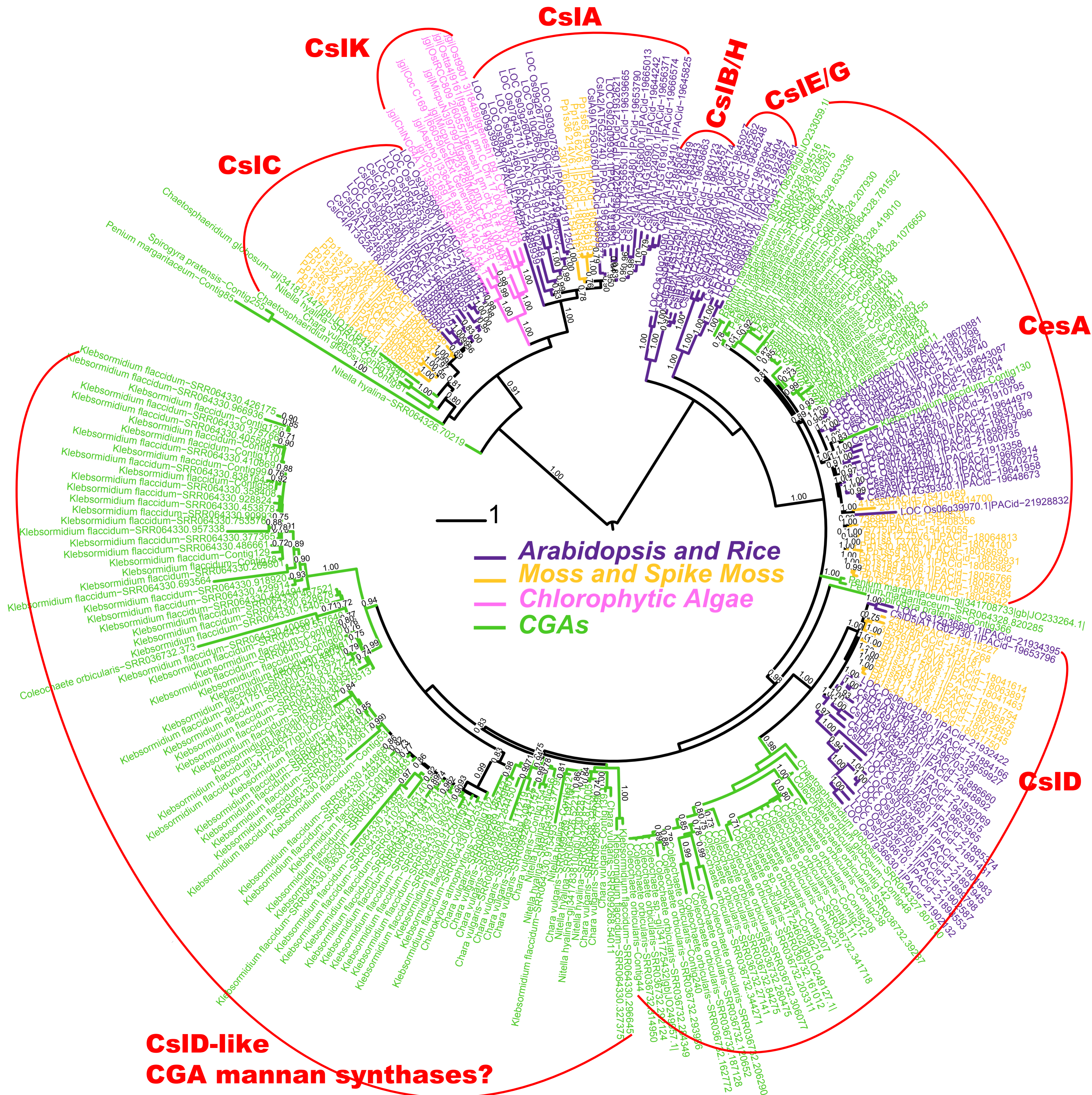

Supplement: Additional file 7 — Phylogeny with CGA homologs longer than 100 a.a. [file 1471-2164-15-260-S7.pdf]
